# Medical Home-Truths.—I

**Published:** 1917-01-13

**Authors:** 


### MEDICAL HOME-TRUTHS.—I.

THOSE interested in antinomy will have noticed, any time this last two years, but more and more of late, that example of it so apparent in our war-time talk and writing. On the one hand, there is complacence, confidence, favourable self-criticism, "optimism," which in a combatant country characterise most the *décor* of discussion of war matters. On the other—if in lesser volume—there presents itself some telling of disagreeable truths. For instances of the former, take almost any railway carriage, almost any newspaper: of the latter, Lord Selborne's report (as to the relative efficiency of English and of German farmers), or the book just published entitled "Eclipse or Empire?" or Mr. Filson Young's opening article in a recent number of a certain review.

And so, a lead having been given in the spheres of agriculture, of commerce and industry, and of general intellectual affairs, why should not the examination in question be extended to medicine? If, as seems probable, there were illusions in existence having relation to them, and needing to be destroyed, it might be well to see if there are any relating to British medicine. One fears that there are; and for once let us tell of them. Lord Selborne set a good example in the plain businesslike ruthlessness of his treatment of the subject, and we will endeavour to be, like him, concrete and definite.

On the writer's shelf stand five separate publications, which are all concerned with one department of medicine. Three of them are German. The first is a monthly *Centralblatt*, that is a medium of record of all appertaining literature in every country. Short summaries and reviews, in either the German, the French, or the English language, are given of every article and book with any claim to be noteworthy. And if Austro-German work bulks as largely as all the others put together, this is not from its being given disproportionate mention. The second periodical is a *Zeitschrift*, a larger and fuller journal, principally devoted to original articles, and also appearing monthly. In the copy at hand, the first of these articles is by an American worker of merit, and is printed in full in English. (What British medical paper ever prints anything by a foreigner in his own language save by the rarest chance?) The third periodical deals only with clinical aspects of the subject. Its high standard of publication is on a par with that of the other two. To continue, numbers four and five are French. The former, the *Revue*, stands very little indeed below the *Zeitschrift* aforementioned; it likewise appears every month. If not so fully, it, too, carefully collates, at the inevitable expense of much time and trouble, a large amount of foreign literature at the end of the original articles. All these five publications are devoted to scientific matter: the inside of the cover, perhaps one leaf in addition, contain advertisements, and these mainly publishers' announcements. Now, number six is the English quarterly. If one were to cut out the subject-matter proper, which is less than that given in any of the above, the excised letterpress would hardly outweigh the pages of advertisements of proprietary articles. Indeed, the review columns tail off into editorial puffs of such, even down to things like fountain-pens and butterscotch. With few exceptions the articles are slight, cut down, snippety and cross-headed. All literature except that easiest of access is unmentioned. These three countries, England, France, and Germany, are the only ones producing special journals on this branch of medicine. America, perhaps because of the difficulties of inter-State circulation, has none.

But to generalise from one instance is fallacious; let us take another. It need not detain us long. The same judgment must be passed, although not so emphatically. But who, after all, could compare that impressive *Archiv*, that earnest *Revue hebdomadale*, with this slight, formal, and perfunctory *Journal?*

Even so, says the necessary and welcome critic, granting you that the Continentals are better than the Anglo-Americans at mere bookmaking and discussion, how stands the balance as regards practical knowledge and mastery of the subject? A pertinent question, because much of medical as of any technical literature is worthless, publicity merely helping to thresh out the corn from the chaff. But the correct answer to it does not involve any change in the tenor of these remarks: of three principal remedies for the disease first noticed one is solely, one almost solely, and the last partially of German origin. Many of its physical signs were first described by the French, who also devised the chief of the current methods by which its administrative control is attempted. As regards the second specialty, the two main therapeutic advances of recent years came from Germany, one of them owing a little also to the enterprise of American workers.

So much for comparative efficiency in two medical specialities. In the ensuing article we pass with some diffidence (for no one man nowadays can review the whole of medicine) to a like examination of the state of things in medicine in general. What has been given up to the present, however, is a fair selection of disagreeable facts—facts which require facing just as much as do the amazing figures of international competition courageously revealed by Dr. Gray and Mr. Turner in their book, and by the Cabinet Minister in his report.

